# Unconventional Imaging Methods in Psoriatic Arthritis

**DOI:** 10.1007/s11926-024-01174-5

**Published:** 2025-01-10

**Authors:** Ioanna Minopoulou, Filippo Fagni, Armin Atzinger, Fredrik Albach, Georg Schett, Gerhard Krönke, Arnd Kleyer, David Simon

**Affiliations:** 1 Department of Rheumatology and Clinical Immunology, Charité - Universitätsmedizin Berlin, Berlin, Germany; 2 Department of Internal Medicine 3, Friedrich-Alexander-University Erlangen-Nürnberg (FAU) and Universitätsklinikum Erlangen, Erlangen, Germany; 3 Deutsches Zentrum Immuntherapie (DZI), Friedrich-Alexander-University Erlangen-Nürnberg (FAU) and Universitätsklinikum Erlangen, Erlangen, Germany; 4 Department of Nuclear Medicine, Friedrich-Alexander-University (FAU) Erlangen-Nürnberg and Universitätsklinikum Erlangen, Erlangen, Germany

**Keywords:** Metabolic imaging, Psoriatic arthritis, Positron emission tomography, Optoacoustic imaging, Artificial intelligence

## Abstract

**Purpose of Review:**

Psoriatic arthritis (PsA) is a complex heterogeneous inflammatory disease that affects about one-third of patients with psoriasis. PsA leads to significant physical impairment and reduced quality of life. Therefore, early diagnosis and intervention are critical for improving long-term outcomes. The purpose of this review is to highlight the advantages of unconventional imaging methods in the diagnosis and management of PsA and to discuss recent advancements in imaging technology.

**Recent Findings:**

Conventional imaging methods, such as radiography, musculoskeletal ultrasound, and magnetic resonance imaging, have been instrumental in detecting structural joint damage and inflammation. However, these imaging modalities have several limitations, resulting in their inability to detect early disease changes. Recent advancements in imaging technology have led to the development of novel imaging modalities capable of characterizing not only early structural but also molecular aspects of disease activity. These cutting-edge approaches have been lately applied to both psoriasis and PsA patients, offering new insights into disease progression, the transition from psoriasis to PsA, and treatment responses.

**Summary:**

By providing more detailed and individualized assessments, unconventional imaging modalities may bring us closer to realizing the potential of personalized medicine in the management of PsA.

## Introduction

Psoriatic arthritis (PsA) is a complex inflammatory condition affecting approximately one-third of patients with psoriasis. Clinical manifestations are diverse, ranging from enthesitis, and dactylitis to peripheral arthritis or axial involment (spondylitis), resulting in heterogeneous disease phenotypes (1). PsA carries a substantial disease burden, leading to reduced physical function and quality of life (2). Hence, early diagnosis and timely treatment initiation are critical to improving clinical outcomes.

However, the clinical heterogeneity of PsA and the lack of specific disease biomarkers make prompt diagnosis and management particularly challenging. In fact, it is estimated that up to 15% of psoriasis patients followed in dermatology clinics have undiagnosed PsA, and typically more than a year elapses from symptom onset until confirmation of diagnosis (3, 4). These diagnostic delays are associated with worsening physical function and accelerated radiographic progression (4, 5). Furthermore, the observed low response rates to treatment in multiple randomized controlled trials and cohort studies despite active treatment with potent disease modifying antirheumatic drugs (DMARDs) such as TNF-, IL-23-, IL-17 or JAK inhibitors, underscore the unmet clinical needs in PsA management (6).

To address these challenges, novel approaches are essential for providing a detailed characterization of this complex disease. Imaging has already proven crucial in the diagnosis and management of PsA. For instance, although no diagnostic criteria exist for PsA, evidence of new bone formation in conventional radiography is included to the CASPAR classification criteria, highlighting the role of imaging as a valuable adjunct in managing PsA (7). Beyond conventional radiography, other modalities such as musculoskeletal ultrasound (MSUS) and magnetic resonance imaging (MRI) have also expanded our understanding of psoriatic disease. Despite these advancements, each technique has limitations. Conventional radiography detects osteoproliferative and erosive disease only after chronic, irreversible damage has occurred. Although MSUS and MRI can sensitively detect soft tissue inflammation and structural changes, they offer a limited field of view and resolution, and scanning of multiple regions is time-consuming (8).

Therefore, while current imaging methods contribute significantly to PsA diagnosis and management, there is a clear need for further innovation in this area to overcome these limitations and improve patient outcomes. Within the past few decades, advanced imaging techniques have emerged, enabling not only the visualization of structural changes but also the molecular characterization of disease processes (8, 9). Several of these technologies have already been applied in psoriasis and PsA patients, showing promise in enhancing our understanding of psoriatic disease. These techniques hold potential for addressing critical aspects such as the progression from psoriasis to PsA, disease flares and treatment response, thereby bringing the goal of personalized medicine closer to clinical reality.

Herein, we present the main examples of unconventional imaging modalities including high-resolution peripheral quantitative CT, positron emission tomography, fluorescence optical imaging and optoacoustic imaging and discuss the current evidence and their implications in the management of PsA. In addition, we briefly address the role of artificial intelligence in the imaging of PsA and its potential in integrating unconventional imaging techniques in the current PsA diagnostic and management framework.

## High-Resolution Peripheral Quantitative CT

High-resolution peripheral quantitative CT was originally developed to investigate bone diseases such as osteoporosis and osteopenia. This imaging instrument has various advantages compared to conventional imaging methods such as X-Ray. HR-pQCT can be used to measure volumetric bone mineral density (vBMD) and bone microstructure, with the particular benefit that vBMD of the trabecular and cortical compartments can be assessed separately. In addition, it enables in vivo bone strength measurement using finite-element analysis. With its high-resolution even small structural catabolic (erosive) and anabolic (osteoproliferative) bone changes can be detected, localized, quantified and monitored with high sensitivity. Therefore, HR-pQCT was increasingly used to analyze bone structures in small joints affected by conditions such as rheumatoid arthritis, arthritis urica, axial spondyloarthritis, osteoarthritis and PsA (10–23).

HR-pQCT has revolutionized our grasp of PsA by unveiling how mechanoinflammation operates at a deeper tissue level. Originally associated with the Koebner phenomenon, where psoriasis develops at skin trauma sites, this mechanism is now understood to affect also the bony compartment of the synovio-entheseal complex (SEC) of patients with psoriatic disease. Mechanical stress in genetically predisposed patients triggers pathological changes in deeper tissues like the SEC. This results in abnormal anabolic bone growth at entheseal sites and the development of the characteristic structural entheseal lesions (SEL). This proliferative process, which is triggered by mechanical stress in deep tissue, can be referred to as the “deep Koebner phenomenon”. Various HR-pQCT studies have identified that (i) SEL can appear in psoriasis patients without any signs of PsA (12) (arthritis, dactylitis, enthesitis, axial involvement), highlighting a potential early marker for PsA and (ii) that these lesions are more frequent in psoriasis patients compared to healthy controls and are associated with a higher risk of developing PsA (11). Interestingly, these lesions correlate with disease duration and negatively impact physical function, emphasizing the importance of early intervention to prevent irreversible damage (23).

HR-pQCT data also suggest that targeted therapies, for example with IL-17 inhibition, can effectively stabilize erosive and osteoproliferative changes in PsA (19, 24). Likewise, IL-17 inhibition effectively prevented the progression of microstructural damage in psoriasis patients with a high risk of developing psoriatic arthritis (PsA) (25). Additionally high-resolution CT studies indicate that biological DMARDs can improve bone strength and mass in PsA patients compared to conventional synthetic DMARDs or no DMARDs, even when disease duration is longer (13).

However, HR-pQCT faces significant challenges in clinical settings due to its inability to detect inflammation and its limited field of view, which confines it to imaging peripheral bones. Despite these limitations, HR-pQCT remains a crucial research tool, significantly advancing our knowledge of bone pathology and treatment responses in PsA. The development of next-generation scanners, such as the Xtreme CT Scanner II, along with improvements in outcome standardization through the SPECTRA collaboration, promises to enhance its research capabilities and impact in the future (26).

## Fluorescence Optical Imaging

During inflammatory processes, microcirculatory changes are marked by vasodilation, increased vascular permeability and neoangiogenesis. Fluorescence optical imaging (FOI) is a novel technique that enables the visualization of these changes in the wrist and finger joints. In particular, FOI involves the intravenous administration of a fluorescent contrast agent, which accumulates more significantly in inflamed tissues than in healthy ones due to the enhanced microcirculation. When exposed to near-infrared light, the fluorescent agents emit signals that are quantified and visualized using a specialized camera, allowing for the precise detection of inflammation.

FOI has proven effective in visualizing synovitis and tenosynovitis in patients with inflammatory arthritis, correlating well with clinical disease activity, musculoskeletal ultrasound and MRI findings. In the context of PsA, various studies have highlighted FOI’s potential in detecting early signs of the disease. In one study involving patients with confirmed and suspected PsA, FOI demonstrated greater sensitivity in detecting inflammation of the DIP and PIP joints compared to musculoskeletal ultrasound. Additionally, FOI was able to distinguish between patients with suspected and confirmed PsA (27). These results were later validated in a follow-up study, where patients who transitioned from suspected to confirmed PsA exhibited an increase in the number of joints with pathological enhancement in the FOI (28). Similar results were reported in a recent multicenter longitudinal study including 389 patients with psoriasis and musculoskeletal complaints and/ or nail psoriasis who underwent clinical examination, FOI and musculoskeletal ultrasound. 77 patients were diagnosed only with psoriasis, while PsA was diagnosed in 140 and additional 55 patients through clinical examination alone and combination of clinical examination and musculoskeletal ultrasound respectively. The remaining 116 patients were only FOI-positive and underwent additional MRI assessment, with 40% of these patients showing positive findings both in FOI and MRI. During a two-year-follow-up, clinical PsA was confirmed in additional 8 only FOI-positive patients, underlining FOI’s potential in the early detection of psoriasis patients at risk for transitioning to PsA (29).

FOI has also revealed distinct inflammatory patterns between RA and PsA. More specifically, while both disease groups exhibited increased signal intensity in affected joints, only PsA patients displayed a unique triangular enhancement extending from the nail bed into the distal interphalangeal joint. This characteristic enhancement, referred to as the “green nail” phenomenon, was specific to PsA, and could therefore represent a potential screening tool for identifying psoriasis patients who are at increased risk of developing PsA (30). However, large-scale prospective studies involving Psoriasis patients without evident PsA symptoms are required to validate its effectiveness.

Despite its potential, FOI has certain limitations. It is an invasive procedure requiring fluorescent dye, which may be contraindicated in patients with liver or kidney impairments or those with allergic reactions. Additionally, increased skin pigmentation can interfere with the accuracy of the results (31). Furthermore, FOI provides a non-specific measurement of microcirculation limited to the joints of the hands and wrists, which restricts its broader application in clinical settings.

## Positron Emission Tomography

Positron emission tomography (PET) is an established molecular imaging technique widely used to assess inflammatory conditions. PET-CT provides detailed information about active lesions across the body in a single scan, offering an advantage over methods like MRI and ultrasound, which have limited fields of view. Fluorine-18 fluorodeoxyglucose (^18^F-FDG) PET-CT, which uses a glucose analogue tracer, was initially developed for cancer imaging but is now experimentally used to detect inflammation in PsA. ^18^F-FDG imaging was able to detect both active disease and subclinical arthritis, facilitating earlier diagnosis (32–34). Recent data also suggest that ^18^F-FDG PET could serve as a potential marker of disease activity allowing for monitoring of treatment response in PsA patients (35).

**Fig. 1:**
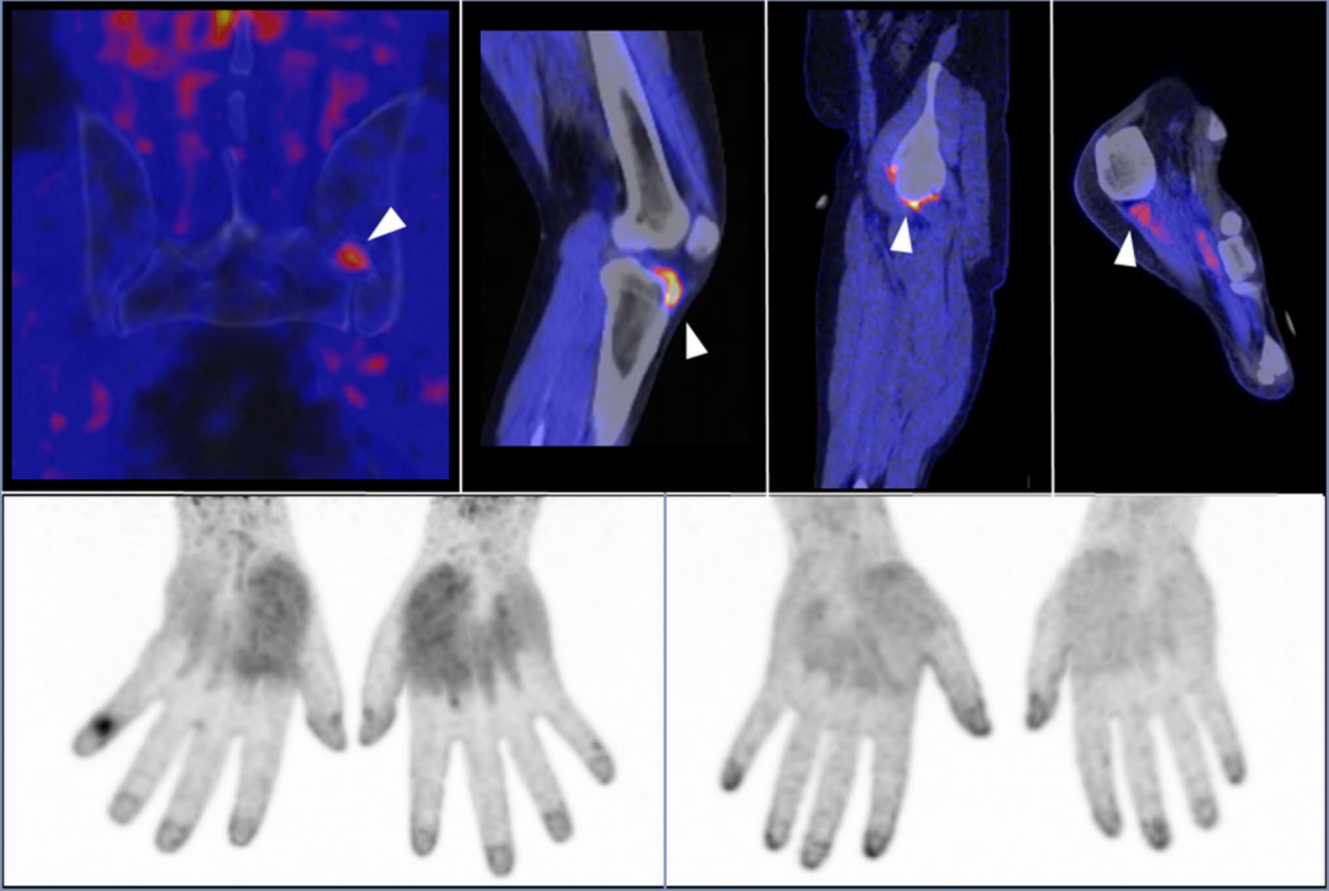
^68^ Ga-FAPI-PET/CT imaging of patients with active Psoriatic Arthritis. Upper panel (left to right): sacroiliac joint FAPI uptake in a PsA patient with sacroiliitis, FAPI-positive synovitis with effusion in the knee joint, synovial FAPI uptake in the hip joint during monolateral coxitis, enthesitis of the plantar fascia with perientheseal FAPI uptake. Lower panel (left to right): DIP joint arthritis of the 5th right finger with correspondent FAPI signal increase, FAPI tracer accumulation at the periungual region of all fingers in the context of active nail psoriasis

Recently, a groundbreaking 68-gallium-labeled radionuclide targeting fibroblast activation protein (FAP) has been developed and approved for clinical use (36). This tracer, known as 68-gallium-labeled FAP inhibitor (^68^ Ga-FAPI-04), enables the visualization of activated fibroblasts in vivo using PET-CT, providing unparalleled insights into mesenchymal tissue activation across the entire body. The technology has already been successfully applied in several autoimmune diseases showing demonstrating its potential to reveal tissue remodeling and inflammation (37) (38). A recent prospective cohort study aimed to assess the presence and distribution of activated fibroblasts in the joints and entheses of patients with psoriasis and arthralgia – but without any signs of arthritis- and to evaluate the risk of developing PsA over time using ^68^ Ga-FAPI-04 PET-CT (39). It was shown, that increased uptake of ^68^ Ga-FAPI-04 was predominantly observed in large joints and the lower limbs. ^68^ Ga-FAPI-04 signal intensity positively correlated with clinical but not ultrasound findings. Notably, higher ^68^ Ga-FAPI-04 uptake was significantly associated with an elevated risk of developing PsA, regardless of ultrasound findings. This indicates that early changes in the joints and entheses of psoriasis patients transitioning to PsA are marked by activated fibroblasts detectable via the FAP-specific tracer. The presence of a positive ^68^ Ga-FAPI-04 PET/CT signal, highlights its potential as an early marker for PsA, offering a promising avenue for preventing and managing early psoriatic disease (Fig. 1).

Further research with larger, more diverse patient groups is crucial to fully assess ^68^ Ga-FAPI-04 PET-CT's potential. While promising, its effectiveness must be validated across a broader range of inflammatory arthritis as well as osteoarthritis cases. Expanding studies to include varied patient populations will improve our understanding of tissue responses and enhance early detection and disease monitoring. These insights could refine treatment strategies and advance the management of PsA.

## Optoacoustic Imaging

Optoacoustic imaging, or photoacoustic imaging, encompasses a group of imaging techniques that enable non-invasive in vivo assessment of tissue molecules by leveraging the optoacoustic effect. More specifically, the optoacoustic effect involves the generation of acoustic waves from tissue molecules following the absorption of ultrashort light pulses which induces thermoelastic expansion. The emitted acoustic waves can be subsequently detected by an ultrasound transducer, allowing detailed imaging and analysis of tissue composition.

Multispectral optoacoustic tomography (MSOT) is an advanced hybrid technology that combines ultrasonography with optoacoustic imaging. By illuminating tissues with near-infrared lasers at multiple wavelengths, MSOT facilitates quantification of endogenous chromophores such as deoxyhemoglobin, oxyhemoglobin, lipids, and collagen based on their specific absorption characteristics. This enables the detection of metabolic changes in tissues and their precise localization through the high spatial resolution offered by ultrasound (8, 9). Therefore, MSOT’s ability to integrate molecular and anatomical data holds significant potential to provide insights into the pathophysiological processes underlying inflammatory diseases.

**Fig. 2:**
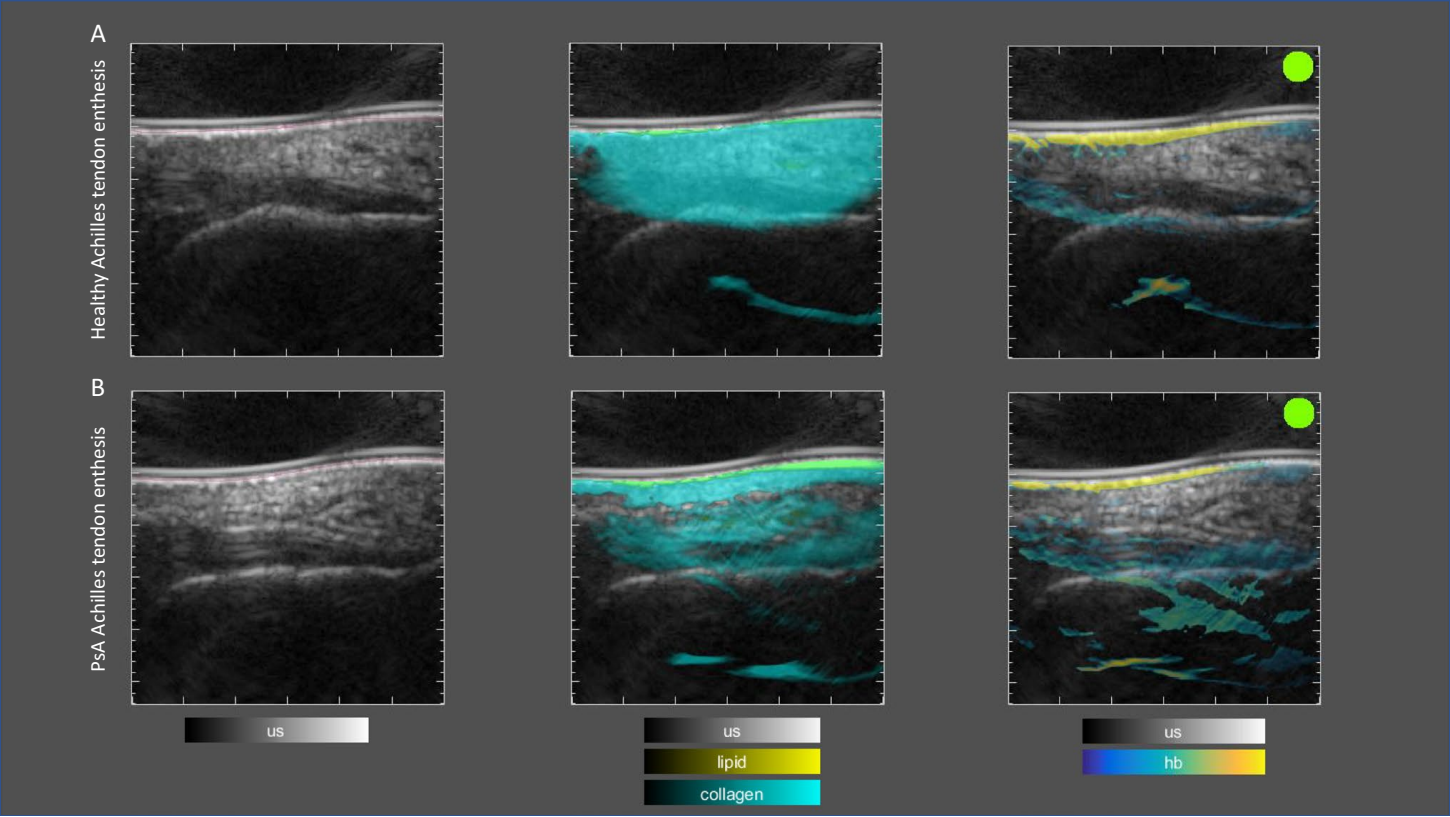
MSOT imaging of the Achilles tendon entheses of a patient with active PsA. The images show normal findings in the left Achilles tendon (A, upper panels) and signs of enthesitis of the right Achilles tendon (B, lower panels). The healthy enthesis shows normal ultrasound findings (left panel), homogeneous collagen distribution (middle panel), and no visible increase in hemoglobin (right panel). The inflamed enthesis (B, lower panels) was clinically tender, swelling was visible on ultrasound (left panel), collagen was reduced and inhomogeneously distributed (middle panel), hemoglobin was diffusely increased and especially at the entheseal site (right panel). US: ultrasound; hb: hemoglobin

Currently limited data are available regarding the applicability of MSOT in PsA. MSOT has been utilized for the assessment of arthritis in the hands of patients with suspected or confirmed PsA as well as in healthy controls (40). PsA patients exhibited higher levels of oxyhemoglobin and deoxyhemoglobin in the finger joints compared to healthy individuals, suggesting impaired oxygen saturation in the inflamed synovial tissues. Additionally, MSOT has revealed distinct metabolic profiles for synovitis and enthesitis. In particular, enthesitis was characterized by increased hemoglobin, oxygen saturation, and collagen content, indicating enhanced vascularization and collagen deposition, likely due to the expansion of mineralized fibrocartilage in calcified entheses (Fig. 2) (41). In contrast, synovitis displayed elevated hemoglobin levels but lower oxygen saturation and collagen content, which aligns with increased metabolic activity in inflamed synovium and collagen degradation. Another MSOT study, the so-called MAPSA trial, investigated metabolic changes at various entheses (lateral humeral epicondyle, distal patellar tendon attachment, and Achilles tendon attachment) in patients with psoriasis (*n* = 30) and PsA (*n* = 30) and compared them to healthy participants (*n* = 30) (42). Both disease groups showed increased vascularization and collagen degradation, with these changes being more pronounced in PsA patients compared to those with psoriasis. Notably, site-specific differences were observed, with the Achilles tendon showing the greatest discrepancies between the healthy controls and the psoriasis and PsA group, supporting the concept of mechanoinflammation. Overall, the MAPSA trial, provided valuable insights, demonstrating that MSOT may be effective in identifying early entheseal inflammation in psoriasis and PsA patients. The results support the concept of a "psoriatic syndrome" spanning from psoriasis to PsA, with MSOT potentially serving as a tool for detecting early enthesitis and assessing the risk of PsA progression in at-risk psoriasis patients.

These findings highlight the promising role of MSOT in revealing metabolic changes associated with psoriatic disease. In the future, MSOT could be instrumental for identifying imaging markers that predict treatment outcomes and in non-invasively monitoring therapeutic efficacy at the tissue level. However, to maximize its potential, further advancements are necessary, including standardization of measurements and conduction of validation studies.

## Role of Artificial Intelligence in the Imaging of PsA

The future of imaging, particularly with the integration of artificial intelligence (AI) and neuronal networks, holds immense promise for revolutionizing the diagnosis and management of various medical conditions, including psoriatic disease. AI-powered imaging technologies are expected to enhance the accuracy and efficiency of disease detection by automating complex tasks such as image analysis, pattern recognition, and data interpretation. Neural networks, with their ability to learn from vast amounts of data, can uncover subtle disease-specific patterns that may go unnoticed by human observers, leading to earlier and more precise diagnoses. As these technologies continue to evolve, we can anticipate more personalized treatment approaches, where AI-driven imaging tools help tailor therapies to individual patients based on detailed imaging biomarkers (8). Additionally, the widespread adoption of AI in imaging could significantly reduce healthcare costs and improve access to advanced diagnostic tools, making high-quality care more accessible and efficient.

However, current AI and neuronal network research in PsA is limited, mainly concentrating on disease classification. For instance, AI has been employed in magnetic resonance imaging (MRI) to differentiate between patients with RA, PsA, and psoriasis without clinical signs of PsA (43). 649 MRI scans were evaluated using a neural network, yielding AUROCs of 75% for seropositive RA versus PsA, 74% for seronegative RA versus PsA, and 67% for seropositive versus seronegative RA. Notably, the study also found that neural networks frequently classified psoriasis patients without PsA as having PsA, implying the presence of PsA-like MRI patterns early in the disease's progression. Another imaging study utilized neural networks to analyze 932 high-resolution CT-derived images of hand joints from patients with RA, PsA, and healthy individuals, aiming to classify inflammatory arthritis (44). This method pinpointed disease-specific joint regions, which were visualized as heat maps, revealing PsA-specific entheseal hotspots. The network effectively differentiated between disease classes, with AUROCs of 82% for healthy individuals, 75% for RA, and 68% for PsA.

Given the clinical significance of nail disease in PsA and psoriasis, along with its treatment challenges, accurate, rapid, and user-independent quantification and monitoring are crucial. Interestingly, a neural network research initiative used hand images from patients with psoriasis (without any signs of PsA) and PsA to automatically quantify nail disease. This network achieved an AUROC of 88%, with a high Pearson correlation (90%) between human annotations and network predictions at the patient level (45). Such advancements in imaging could reduce the frequency of clinic visits and associated costs while facilitating more remote evaluations of psoriatic nail damage.

A recent study aimed to explore the automated scoring of bone erosions, osteitis, and synovitis using MRI data of PsA and RA patients using a deep learning-based convolutional neural network (CNN) approach (46). The CNN achieved high accuracy, with macro-AUC scores of 92% for erosions, 91% for osteitis, and 85% for synovitis. Correlations with human assessments were strong: 90% for erosions, 78% for osteitis, and 69% for synovitis. Taken together these data demonstrated a high potential for future clinical applications, offering a rapid, accurate, and efficient method to assess inflammatory changes in hand MRIs of PsA patients. This approach could lead to more standardized, cost-effective evaluations in clinical settings.

However, the current AI findings in PsA remain preliminary. To fully integrate AI into clinical settings, enhancing algorithm performance and generalizability is critical. This necessitates large-scale datasets that accurately represent the study population, precise annotations, and external multi-center datasets for independent algorithm validation. Ongoing validation studies, modeled after successful AI implementations in cancer radiology, are crucial. Considering AI's vast potential, its integration into PsA imaging, combined with insights from various imaging modalities, could be a key driver of personalized medicine in PsA management in the future.

## Conclusions

In conclusion, while conventional imaging modalities have significantly contributed to the diagnosis and management of psoriatic arthritis (PsA), their limitations underscore the need for further innovation. The emergence of advanced imaging techniques that enable both structural and molecular characterization holds great promise for improving the understanding of PsA. These technologies offer the potential to detect early disease changes, monitor disease progression, and assess treatment response with greater precision, thereby advancing the field toward personalized medicine. Continued research and development in this area are essential to address the current clinical gaps and ultimately enhance patient outcomes.

## Key Reference

- Simon D, Tascilar K, Kleyer A, Bayat S, Kampylafka E, Sokolova MV, et al. Association of Structural Entheseal Lesions With an Increased Risk of Progression From Psoriasis to Psoriatic Arthritis. Arthritis & rheumatology (Hoboken, NJ). 2022;74(2):253-62.
  - Using high-resolution CT, it has been shown that the presence of anabolic changes at the entheses (structural entheseal lesions) is associated with an increased risk of developing PsA in patients with skin psoriasis.

- Simon D, Faustini F, Kleyer A, Haschka J, Englbrecht M, Kraus S, et al. Analysis of periarticular bone changes in patients with cutaneous psoriasis without associated psoriatic arthritis. Annals of the rheumatic diseases. 2016;75(4):660-6.
  - This HR-pQCT study has shed light on important aspects of the so-called “deep Köbner phenomenon” and has shown that the stress response patterns of the joints are similar to those of the skin (psoriatic plaques).

- Corte G, Atzinger A, Temiz SA, Noversa de Sousa R, Mutlu MY, Schoenau V, et al. Anatomical pattern of entheseal and synovial fibroblast activation in patients with psoriasis and its risk of developing psoriatic arthritis. RMD Open. 2024;10(2).
  - Activation of mesenchymal cells detectable by FAP-specific tracer accumulation represented the earliest change in psoriasis patients progressing to PsA and may precede inflammation, highlighting the potential for targeting mesenchymal cells in the prevention and treatment of early psoriatic disease.

- Fagni F, Tascilar K, Noversa de Sousa R, Bayat S, Sollfrank L, Kleyer A, et al. Unveiling Metabolic Similarities of Entheses in Patients with Psoriasis and Psoriatic Arthritis Using Noninvasive In Vivo Molecular Imaging: Results From a Cross-sectional Exploratory Study. Arthritis Rheumatol. 2024;76(9):1387-96.
  - Psoriasis patients, both with and without arthritis, showed different metabolic entheseal patterns that were not seen in healthy individuals using optoacoustic imaging, suggesting that this novel imaging technique could detect early enthesitis and aid in the assessment of progression from psoriasis to PsA.

- Folle L, Fenzl P, Fagni F, Thies M, Christlein V, Meder C, et al. DeepNAPSI multi-reader nail psoriasis prediction using deep learning. Sci Rep. 2023;13(1):5329.
  - Use of a neural network to classify the severity of psoriatic nail diseases based on nail photos showed the vast potential of artificial intelligence to enhance monitoring of nail psoriasis in clinical trials and remote patient care.

## Data Availability

No datasets were generated or analysed during the current study.
